# Atypical Sensory Processing Profiles and Their Associations With Motor Problems In Preschoolers With Developmental Coordination Disorder

**DOI:** 10.1007/s10578-020-01013-5

**Published:** 2020-06-11

**Authors:** Misaki Mikami, Tomoya Hirota, Michio Takahashi, Masaki Adachi, Manabu Saito, Shuhei Koeda, Kazutaka Yoshida, Yui Sakamoto, Sumi Kato, Kazuhiko Nakamura, Junko Yamada

**Affiliations:** 1 Department of Comprehensive Rehabilitation Science, Graduate School of Health Sciences, Hirosaki University, 66-1 Honcho, Hirosaki, Aomori, Japan; 2 Research Fellow of Japan Society for the Promotion of Science, Tokyo, Japan; 3 Department of Psychiatry, University of California San Francisco, San Francisco, USA; 4 Department of Neuropsychiatry, Graduate School of Medicine, Hirosaki University, Hirosaki, Japan; 5 Department of Clinical Psychological Science, Graduate School of Health Sciences, Hirosaki University, Hirosaki, Japan; 6 Research Center for Child Mental Development, Graduate School of Medicine, Hirosaki University, Hirosaki, Japan; 7 Department of Management and Low, Aomori Chuo Gakuin University, Aomori, Japan

**Keywords:** Developmental coordination disorder, Neurodevelopmental disorders, Sensory processing, Sensory profile, Preschoolers

## Abstract

The aims of this study were to identify sensory processing profiles specific to preschoolers with DCD in a community sample and examine the association of sensory processing problems with motor coordination difficulties in these children. Sixty-three 5-year-old children with DCD and without other neurodevelopmental disorders and 106 age-matched typically developing children participated in this study. Sensory processing problems were assessed using the Sensory Profile. Our results demonstrated problems in wide sensory processing patterns (low registration, sensitivity and avoiding) and areas (auditory, vestibular, touch and oral) in children with DCD compared with typically developing children. Additionally, the association of problems in sensory processing patterns (sensitivity and avoiding) and areas (touch and auditory) with motor coordination difficulties were identified in children with DCD alone. Our findings indicate that sensory processing abnormalities may contribute to the pathophysiology of DCD, suggesting the importance of assessing sensory processing functions in children with DCD.

**Electronic supplementary material:**

The online version of this article (10.1007/s10578-020-01013-5) contains supplementary material, which is available to authorized users.

## Introduction

Developmental coordination disorder (DCD) is a neurodevelopmental disorder primarily characterized by motor coordination impairments, which significantly interfere with activities of daily living and academic performance. The motor coordination difficulties of children with DCD generally occur in the early developmental period and are not explained by intellectual delays, visual impairments, or other neurological conditions that affect movement [1]. The most often reported prevalence of DCD is between 5 and 6% in children but can range from 2 to 20%, depending on the study sample and ascertainment methodologies [1, 2].

Although motor coordination impairments are core symptoms of DCD, this disorder can also lead to non-motor coordination problems such as poor scholastic achievements compared with children without DCD [3, 4]. In addition, children with DCD are less likely to participate in self-care, leisure or physical activity, especially team sports [5, 6]. A relationship has been reported between reduced physical activity and poor self-efficacy [7, 8] and lower life satisfaction [9] in children with DCD.

Several sensory problems co-occur in children with DCD. Previous studies have indicated poor visual-spatial processing skills, proprioception function, hearing and vestibular function in children with DCD [2, 10, 11]. Neuroimaging studies have examined the mechanisms of sensory problems in children with DCD; those studies reported abnormalities in the white matter microstructural organization in the corticospinal tract, posterior thalamic radiation, intraparietal sulcus and parietal subregion of the corpus callosum, areas of the central nervous system that are related to sensorimotor function [12–15].

Although sensory processing profile differences are prevalent in other neurodevelopmental disorders (NDDs), such as autism spectrum disorder (ASD) and attention deficit hyperactivity disorder (ADHD) [16], there have been only a few studies that examined sensory processing profiles in DCD alone. A recent study by Allen & Casey [11] showed that children with DCD but with no other NDDs had sensory processing difficulties, including hearing and body awareness and balance, which were measured by parent-reporting questionnaires. However, the sample in Allen & Casey’s study [11] consisted of clinical samples, limiting the generalization of the study findings to non-clinical samples despite it being considered uncommon for children with DCD alone to present to clinical settings. Additionally, the age of the participants in their study ranged from 5 to 12 years; thus it remains unknown if their study findings apply to different age samples (preschoolers, for example). A previous study reported that sensory processing contributed to motor coordination in 3-year-old children [17]. Therefore, there is a possibility that sensory differences emerge in early developmental stages in children with motor coordination difficulties.

Elbasan et al. [18] reported a correlation between tactile processing ability and fine motor skills in activities of daily living in children with DCD, without excluding ASD and ADHD. Conversely, Allen & Casey [11] reported no correlations between sensory problems and motor skills in children with DCD and co-occurring ASD. However, no studies have examined the association between sensory processing functions and motor coordination skills in children with DCD alone.

Thus, this study aimed to identify sensory processing profiles specific to preschoolers with DCD in a community sample and examine the association of sensory processing problems with motor coordination difficulties in these children. Achieving these aims would deepen our understanding of complex clinical phenotypes in children with DCD and can lead to different approaches/interventions for children who exhibit both motor and sensory function impairments.

## Methods

### Study Design

This is a secondary analysis of data from the Hirosaki 5-year-old developmental check-up (HFC), which is an epidemiological study conducted in 3590 5-year-old children in Hirosaki city from 2016 to 2018 (see Supplemental Figure). The HFC was initiated with the aim of identifying children with NDDs and providing appropriate interventions and accommodations. The HFC comprised two phases: the screening phase and the assessment phase. The developmental screening was conducted using validated screening tools, including the Developmental Coordination Disorder Questionnaire (DCDQ) [19]. Children who screened positive for NDDs were invited to an in-person assessment at the Hirosaki university clinic. The assessment batteries included a child and parent interview, cognitive testing, and motor skills testing using the Movement Assessment Battery for Children, 2nd edition (MABC-2) [20] conducted by licensed occupational therapists and psychologists. More details on the HFC study design was previously published [21]. Additionally, sensory processing patterns were assessed using the Sensory Profile (SP) [22]. For the diagnosis of NDDs, we used the Diagnostic and Statistical Manual of Mental Disorders, 5th edition [1] and the guidelines from the European Academy of Childhood Disability [23]. Each case was discussed among multidisciplinary professionals, including occupational therapists, psychologists, and psychiatrists.

### Participants (Inclusion and Exclusion Criteria)

Participants in the present study were 342 children who attended the in-person assessment. Children with DCD and other co-occurring NDDs (ASD, ADHD, and/or intellectual disability defined as full-scale IQ < 70) were excluded, leaving children with DCD alone and those without any NDDs (defined as typically developing: TD children) as included participants for the present study. We also excluded children that had at least one missing value in each measure.

### Measures

The DCDQ is a 15-item parent questionnaire designed to screen for coordination disorders in children aged 5–15 years [19]. The 15 items are grouped into three distinct factors: ‘Control During Movement’, ‘Fine Motor/Handwriting’, and ‘General Coordination’. The DCDQ has been standardized in Japanese, and the Japanese version of the DCDQ was found to have good psychometrics [24]. In the present study, we used the cut-off scores of the original DCDQ, defined as ≤ 46 [25].

The MABC-2 is designed to assess motor impairments of children aged 3–16 years and comprises eight tasks: three measure manual dexterity, two measure ball skills, and three measure balance [20]. The psychometric properties of the MABC-2 were found to be acceptable overall, with good to excellent reliability, fair to good validity, fair to good sensitivity, and good specificity [26, 27]. Because the MABC-2 has not been standardized in Japanese yet, the original MABC-2 was translated to Japanese (with no back-translation process) by our research and clinical team for use in the developmental checkup. The test was conducted by well-trained occupational therapists, clinical psychologists, experts in developmental psychology.

The SP is a scale used to assess sensory processing. The SP comprises 125 questions covering 14 categories, including six sensory processing areas (auditory, visual, vestibular, touch, multi-sensory and oral sensory) [22]. The SP also includes sensory processing patterns scores, classifying a child’s response and behavior into four types based on the child’s neural threshold (high or low) and behavioral strategies to the sensory information (active or passive), which include low registration, sensation seeking, sensory sensitivity, and sensation avoiding. Higher score indicates that the child has more behaviors associated with sensory processing problems. The SP was standardized in Japanese [28], and showed comparable psychometric properties with the original SP. In the present study, caregivers (primarily parents) reported each item of the SP on a five-point Likert scale.

We used other tests as covariates. We used the Wechsler Intelligence Scale for Children, 4^th^ edition (WISC4) [29, 30] full-scale IQ to assess cognitive ability. We also used the total score from the Social Responsiveness Scale, 2^nd^ edition (SRS2) [31, 32] and the global index score from Conners' Third Edition Parent Rating Scale (Conners 3) [33, 34] to assess ASD and ADHD traits, respectively. We used the Japanese version of these tests.

### Analytic Plans

For demographic data, we examined the difference in sex ratio between children with DCD and TD using the chi-squared test. We also performed a t-test to examine the differences in age, full-scale IQ, SRS2 total score, and the Conners3 global index score between two groups.

To compare the total and subscale scores on the MABC-2 and the DCDQ between two groups, we performed multiple analysis of covariance (MANCOVA) on the total and subscale scores on the MABC-2 and the DCDQ between two groups. Sex and the full-scale IQ were used as covariates to control the influence of possible confounding factors. When a significant main effect between two groups was observed in MANCOVA, we conducted one-way analysis of covariance (ANCOVA) specifying sex and the full-scale IQ as covariates to examine the differences in the total and subscale scores on the MABC-2 and DCDQ between two groups.

The SP subscale scores were compared using two-way ANCOVAs, with two groups (TD or DCD) as a between-group factor and the SP four sensory processing patterns or six areas as a within-subject factor. Sex and the full-scale IQ were used as covariates to control the influence of possible confounding factors. When a significant main effect and/or interaction related to two groups was observed in two-way ANCOVA, we conducted one-way ANCOVA specifying sex and the full-scale IQ as covariates to examine the differences in the SP subscale scores between two groups. A partial η^2^ was reported as the effect size for these analyses.

We then performed stepwise multiple regression analysis in each group to examine whether sensory processing problems were associated with motor coordination difficulties beyond the possible confounding demographics (sex and the full-scale IQ) and whether the associations were specific to children with DCD.

SPSS version 24.0 was used to perform all analyses. The level of statistical significance was defined as *p* < 0.05.

## Results

### Participants

Among 342 children who attended the in-person assessment, 227 were diagnosed with NDDs (DCD: 151, ASD: 70, ADHD: 101, intellectual disability: 70). Table 1 presents the demographic characteristics and neurodevelopmental disorder symptoms of the participants, including 63 children with DCD and 106 TD children. The ratio of boys was significantly higher in the DCD group than in the TD group, and the full-scale IQ in the DCD group was significantly lower than that in the TD group. No significant differences in age, the SRS2 total score, or the Conners3 global index score were identified between two groups.

**Table 1:** Demographic participants information

	DCD (n = 63)		TD (n = 106)		Analysis	
	M	SD	M	SD	χ^2^/*t*	*p*
Sex (boy: girl)	43: 20		54: 52		4.84	.028
Age (months)	64.1	1.7	64.1	1.9	− 0.20	.842
Full-scale IQ	88.5	10.2	98.2	12.3	5.25	< .001
SRS2 Total	37.0	17.9	32.4	16.4	− 1.69	.092
Conners3 Global Index	8.0	4.0	7.5	4.8	− 0.71	.478

*DCD* developmental coordination disorder, *DCDQ* developmental coordination disorder questionnaire, *Full-scale IQ* full-scale intelligence quotient, *MABC-2* movement assessment battery for children second edition, *TD* typically developing

Significant main effects between two groups were observed in MANCOVA on the total and subscale scores on the MABC-2 (*F*_(4, 162)_ = 43.62, *p* < 0.001, η_p_^2^ = 0.52) and DCDQ (*F*_(3, 163)_ = 9.64, *p* < 0.001, η_p_^2^ = 0.15). Table 2 shows the results of one-way ANCOVAs on the total and subscale scores on the MABC-2 and DCDQ. The DCD group had lower scores on all MABC-2 and DCDQ total and control during movement subscales compared with the TD group.

**Table 2:** Differences of total and subscale scores on the MABC-2 and the DCDQ between DCD and TD group

	DCD (n = 63)		TD (n = 106)		ANCOVA		
	*M*	*SD*	*M*	*SD*	*F*	*p*	η_p_^2^
MABC-2							
Total	5.6	1.7	10.2	2.0	176.69	< .001	.52
Manual dexterity	5.9	2.4	10.0	2.4	77.76	< .001	.32
Aiming & catching	6.6	2.6	9.8	2.5	59.25	< .001	.26
Balance	7.4	2.1	10.7	2.5	52.63	< .001	.24
DCDQ							
Total	43.4	9.7	50.8	9.6	17.54	< .001	.10
Control during movement	17.1	4.6	20.9	4.6	28.34	< .001	.15
Fine motor/handwriting	12.2	3.9	14.2	3.3	5.75	.018	.03
General coordination	14.1	3.7	15.6	3.7	4.33	.039	.03

*ANCOVA* analysis of covariance, *DCD* developmental coordination disorder, *DCDQ* developmental coordination disorder questionnaire, *MABC-2* movement assessment battery for children second edition, *TD* typically developing

### Comparison of Sensory Processing Functions Between the Groups

**Fig. 1:**
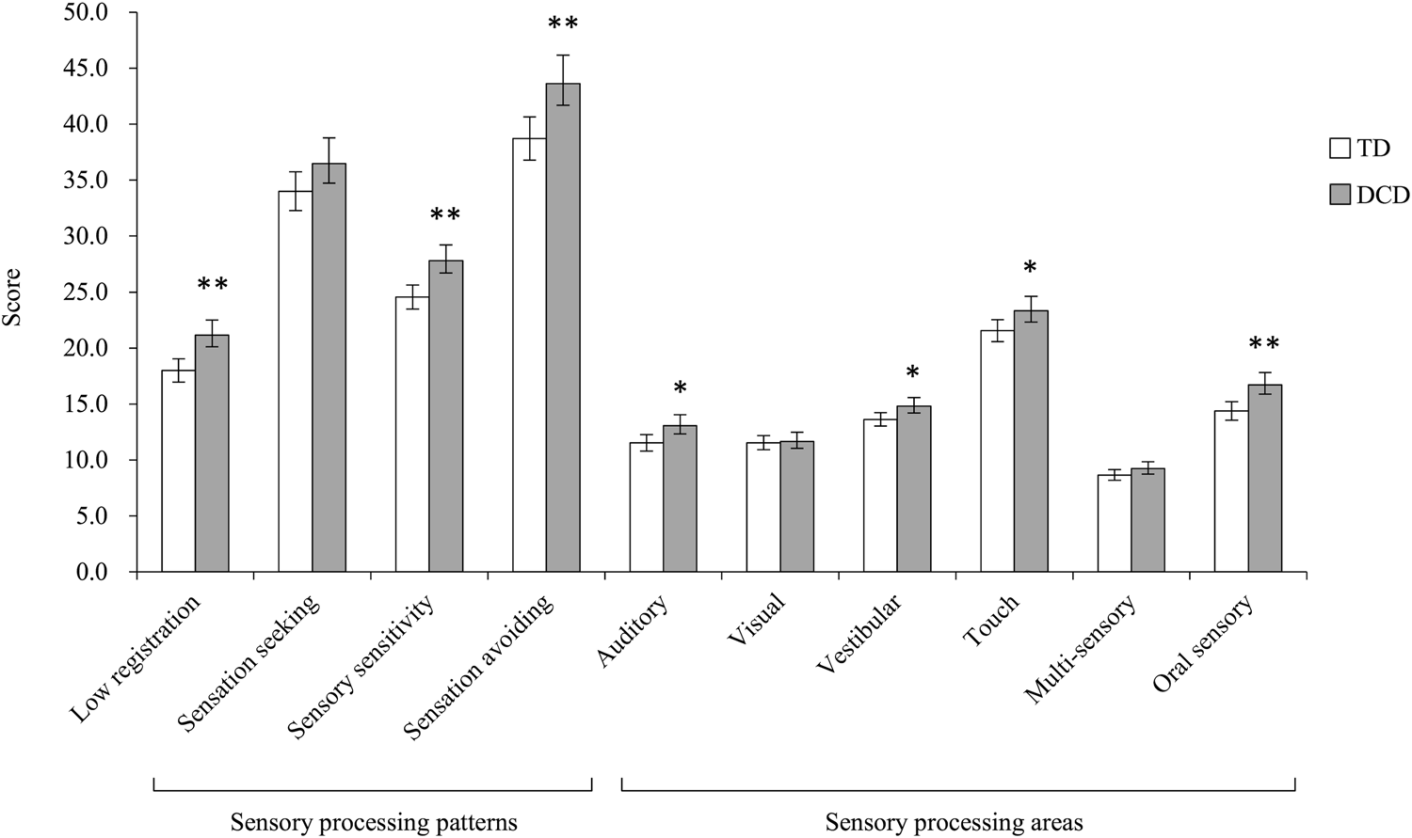
Differences of SP sensory processing patterns and areas scores between the DCD group and the TD group. Number of sample in each group are as follows: DCD group (*n* = 63); TD group (*n* = 106). Each column shows SP sensory processing patterns and areas scores and error bar represent 95% confidence interval. The results of one-way analyses of covariance are as follows: Low registration, *F*_(1, 165)_ = 12.29, *p* = .001, η_p_^2^ = .07; Sensation seeking, *F*_(1, 165)_ = 2.68, *p* = .104, η_p_^2^ = .02; Sensory sensitivity, *F*_(1, 165)_ = 11.73, *p* = .001, η_p_^2^ = .07; Sensation avoiding, *F*_(1, 165)_ = 8.52, *p* = .004, η_p_^2^ = .05); Auditory, *F*_(1, 165)_ = 5.63, *p* = .019, η_p_^2^ = .03; Visual, *F*_(1, 165)_ = 0.05, *p* = .825, η_p_^2^ < .01; Vestibular, *F*_(1, 165)_ = 5.12, *p* = .025, η_p_^2^ = .03; Touch, *F*_(1, 165)_ = 4.16, *p* = .043, η_p_^2^ = .03; Multi-sensory *F*_(1, 165)_ = 1.82, *p* = .179, η_p_^2^ = .01, Oral sensory, *F*_(1, 165)_ = 10.37, *p* = .002, η_p_^2^ = .06). DCD = developmental coordination disorder; TD = typically developing. * *p* < .05, ** *p* < .01

The two-way ANCOVA of the SP scores of sensory processing patterns showed significant main effects of the group (*F*_(1, 165)_ = 11.34, *p* = 0.001, η_p_^2^ = 0.06), whereas an interaction between the group and the SP scores of sensory processing patterns was not significant (*F*_(2.38, 393.36)_ = 1.23, *p* = 0.296, η_p_^2^ < 0.01). The two-way ANCOVA for the SP sensory processing areas showed significant main effects of the group (*F*_(1, 165)_ = 7.12, *p* = 0.008, η_p_^2^ = 0.04). Additionally, there was a significant interaction between the group and the SP sensory processing areas (*F*_(4.05, 667.60)_ = 2.92, *p* = 0.020, η_p_^2^ = 0.02). The results of the one-way ANCOVAs for examining differences in the SP scores are shown in Fig. 1. The DCD group had significantly higher scores than the TD group on three of the four sensory processing patterns (low registration, sensory sensitivity, sensation avoiding) only the sensation seeking score was not significant. In addition, the DCD group has significantly higher scores than the TD group on four subscales of sensory processing areas (auditory, vestibular, touch, oral sensory). These results indicate that children with DCD had difficulties in sensory processing in these areas when compared with those with TD. There was no significant difference in the visual or multi-sensory subscales between the two groups.

### Associations of Sensory Processing Functions with Motor Coordination Skills

Tables 3 and 4 present the results of the stepwise multiple regression analysis with the scores of sensory processing patterns on the SP as explanatory variables to the scores of the MABC-2 and the DCDQ in the DCD group and the TD group, respectively. No significant association was found between the MABC-2 scores and the sensory processing pattern scores. In the DCD group, there were significant negative associations between the DCDQ control during movement score and the SP sensation avoiding score and between the DCDQ fine motor/handwriting score and the SP sensory sensitivity score (Table 3). Additionally, the DCDQ control during movement score was positively associated with the SP sensation seeking score (Table 3). In the TD group, there were a significant negative association between the DCDQ general coordination score and the SP low registration score (Table 4).

**Table 3:** Stepwise multiple regression analysis of scores of the SP sensory processing patterns on scores of the MABC-2 and the DCDQ in the DCD group

	MABC-2						DCDQ					
	Manual dexterity		Aiming & Catching		Balance		Control during movement		Fine motor/handwriting		General coordination	
	β	*p*	β	*p*	β	*p*	β	*p*	β	*p*	β	*p*
Low registration	–	–	–	–	–	–	–	––	–	–	–	–
Sensation seeking	–	–	–	–	–	–	.32	.009	–	–	–	–
Sensory sensitivity	–	–	–	–	–	–	–	–	−.34	.007	–	–
Sensation avoiding	–	–	–	–	–	–	−.42	.001	–	–	–	–
Full–scale IQ	.31	.014	–	–	.44	< .001	–	–	–	–	–	–
Sex	–	–	–	–	–	–	–	–	–	–	−.38	.002
*R*^2^	.095		–		.192		.205		.115		.145	
*p*	.014		–		< .001		.001		.007		.002	

*N* = 63. *DCDQ* developmental coordination disorder questionnaire, *Full-scale IQ* full-scale intelligence quotient, *MABC-2* movement assessment battery for children second edition, − not significant

**Table 4:** Stepwise multiple regression analysis of scores of the SP sensory processing patterns on scores of the MABC-2 and the DCDQ in the TD group

	MABC-2						DCDQ					
	Manual dexterity		Aiming & Catching		Balance		Control during movement		Fine motor/handwriting		General coordination	
	β	*p*	β	*p*	Β	*p*	β	*p*	β	*p*	β	*p*
Low registration	–	–	–	–	–	–	–	–	–	–	−.23	.017
Sensation seeking	–	–	–	–	–	–	–	–	–	–	–	–
Sensory sensitivity	–	–	–	–	–	–	–	–	–	–	–	–
Sensation avoiding	–	–	–	–	–	–	–	–	–	–	–	–
Full-scale IQ	.30	.002	–	–	–	–	–	-	.27	.004	–	–
Sex	–	–	–	–	.26	.007	–	–	–	–	–	–
*R*^2^	.091		–		.067		–		.075		.054	
*p*	.002		–		.007		–		.004		.017	

*N* = 106

*DCDQ* developmental coordination disorder questionnaire, *Full-scale IQ* full-scale intelligence quotient, *MABC-2* movement assessment battery for children second edition, −  not significant

Tables 5 and 6 show the results of stepwise multiple regression analysis with the scores of sensory processing areas on the SP as explanatory variables to the MABC-2 and the DCDQ scores in the DCD group and the TD group, respectively. No significant associations between the MABC-2 scores and the sensory processing pattern scores were found. However, there were significant negative associations between the DCDQ fine motor/handwriting score and the SP touch score and between the DCDQ general coordination score and the SP auditory score in the DCD group (Table 5). Additionally, the DCDQ general coordination score was positively associated with the SP multi-sensory score (Table 5). On the other hand, the DCDQ general coordination score was negatively associated with the SP multi-sensory score in the TD group (Table 6).

**Table 5:** Stepwise multiple regression analysis of scores of the SP sensory processing areas on scores of the MABC-2 and the DCDQ in the DCD group

MABC-2							DCDQ					
	Manual dexterity		Aiming & Catching		Balance		Control during movement		Fine motor/handwriting		General coordination	
	β	*p*	β	*p*	β	*p*	β	*p*	β	*p*	β	*p*
Auditory	–	–	–	–	–	–	–	–	–	–	−.44	.001
Visual	–	–	–	–	–	–	–	–	–	–	–	–
Vestibular	–	–	–	–	–	–	–	–	–	–	–	–
Touch	–	–	–	–	–	–	–	–	−.34	.007	–	–
Multi–sensory	–	–	–	–	–	–	–	–	–	–	.38	.005
Oral sensory	–	–	–	–	–	–	–	–	–	–	–	–
Full-scale IQ	.31	.014	–	–	.44	< .001	–	–	–	–	–	–
Sex	–	–	–	–	–	–	–	–	–	–	−.38	.001
*R*^2^	.095		–		.192		.113		–		.299	
*p*	.014		–		< −001		.007		–		< .001	

*N* = 63

*DCDQ* developmental coordination disorder questionnaire, *Full-scale IQ* full-scale intelligence quotient, *MABC-2* movement assessment battery for children second edition, − not significant

**Table 6:** Stepwise multiple regression analysis of scores of the SP sensory processing areas on scores of the MABC-2 and the DCDQ in the TD group

	MABC-2						DCDQ					
	Manual dexterity		Aiming & catching		Balance		Control during movement		Fine motor/handwriting		General coordination	
	β	*p*	β	*p*	β	*p*	β	*p*	β	*p*	β	*p*
Auditory	–	–	–	–	–	–	–	–	–	–	–	–
Visual	–	–	–	–	–	–	–	–	–	–	–	–
Vestibular	–	–	–	–	–	–	–	–	–	–	–	–
Touch	–	–	–	–	–	–	–	–	–	–	–	–
Multi-sensory	–	–	–	–	–	–	–	–	–	–	−.19	.050
Oral sensory	–	–	–	–	–	–	–	–	–	–	–	–
Full-scale IQ	.30	.002	–	–	–	–	–	–	.27	.004	–	–
Sex	–	–	–	–	.26	.007	–	–	–	–	–	–
*R*^2^	.091		–		.067		–		.075		.037	
*p*	.002		–		.007		–		.004		.050	

*N* = 106

*DCDQ* developmental coordination disorder questionnaire, *Full-scale IQ* full-scale intelligence quotient, *MABC-2* movement assessment battery for children second edition,− not significant

## Discussion

We examined sensory processing profiles specific to preschoolers with DCD diagnosed through methodologically rigorous processes consisting of screening in a general population sample and a subsequent in-person assessment. Although these profiles were previously examined in older children in a clinical sample [11], to the authors’ knowledge, this is the first study that elucidated sensory profiles in preschoolers with DCD using a community sample. The proportion of children with DCD was 4.2% in our sample, and it was comparable to that in the previous reports [2]. Additionally, we examined the association of sensory processing problems with motor coordination difficulties in children with DCD alone. The findings obtained in the present study are novel because we excluded ASD and ADHD, both of which were considered to contribute to atypical sensory profiles in the previous studies that included children with DCD and co-occurring ASD and/or ADHD [11, 18].

### Sensory Processing Profile Specific to Preschoolers with DCD

Our findings revealed atypical sensory profiles in preschoolers with DCD. Using the SP, we found that children with DCD had lower registration (i.e. hypo-responsiveness to sensory stimuli) and more sensory sensitivity and sensation avoiding compared with TD children. Children with DCD also had more sensory challenges in auditory, vestibular, touch and oral areas. Our findings regarding sensory processing patterns have been examined in existing research studies targeting other NDDs. For example, Cascio [35] reported sensory processing abnormalities, specifically hypersensitivity or hyposensitivity to several sensory inputs in individuals with non-DCD NDDs, such as ASD, ADHD, and cerebral palsy. Findings from other existing studies were consistent with those reported in Cascio’s study [35]; for example, a recent study has reported that children with ASD and ADHD have higher trends in all SP sensory processing patterns compared with TD children, respectively [16]. Hyper-reactivity or hypo-reactivity to sensory input or unusual interests in sensory aspects of the environment is now incorporated in the diagnostic criteria for ASD [1]. Although more research is needed, similar to ASD, our findings indicate that sensory processing abnormalities may contribute to the pathophysiology of DCD and thus may need to be considered important diagnostic factors.

Our results showed that children with DCD had problems in broad sensory processing areas, except for visual and multisensory areas. The auditory and vestibular processing problems identified in the present study are in line with those reported in Allen & Casey’s study [11], which showed these sensory processing difficulties measured by parent-reporting questionnaires in 5- to 12-year-old children with DCD. Studies have revealed abnormalities of functional networks involving the cerebellum in DCD [36], and the cerebellum also plays an important role in auditory processing [37]. Therefore, our findings imply that auditory processing (sensory) problems and motor coordination difficulties stem from the same underlying neural mechanism involving the cerebellum.

Abnormalities of tactile and oral sensory processing in ASD have been frequently reported in previous studies [16, 38, 39]. Additionally, Nadon et al. [40] indicated that eating difficulties in ASD likely reflect problems of oral sensory processing. The mechanism accounting for the problem in the oral sensory area in children with DCD has not been previously examined. However, as it is reported that eating challenges and speech/language difficulties could exist in children with DCD in early childhood [41–43], there is a possibility that oral sensory processing problems may affect difficulties involving oral movement in children with DCD. Overall, our findings confirmed that sensory processing challenges widely reported in children with other NDDs, particularly ASD, also existed in ones with DCD alone. These results indicate that sensory processing challenges are not disorder-specific but instead can be transdiagnostic across NDDs, suggesting the possible existence of common underlying mechanisms.

### Associations of Sensory Processing Problems with Motor Coordination Difficulties in Preschoolers with DCD

Our results showed associations between low thresholds in sensory processing (avoiding and sensitivity) and fine and gross motor problems in children with DCD. In addition, the results of multiple regression analysis showed that the association between sensory processing problems and motor coordination difficulties in the DCD group differed from that in the TD group, suggesting that the associations are specific to children with DCD. Compared with other NDDs, research examining the associations between sensory thresholds and motor challenges is limited in DCD. Smits‐Engelsman and Wilson [44] have suggested that excessive sensory noise, which is one of the neural noises in the motor system, is associated with poor motor prediction and makes the problem of motor control more difficult in DCD. Another study revealed correlations between the SP sensory sensitivity and motor skills in daily activities in 5- to 13-year-old children with NDDs [45]. However, these findings were inconsistent with those reported in a study examining the association of tactile thresholds with fine motor difficulties in children with ADHD [46]. Puts et al. [46] reported associations between high tactile thresholds in sensory processing and fine motor problems in children with ADHD and suggested that high detection thresholds may reflect the impaired perception of relevant information above the noise. Moreover, the lack of awareness of tactile information could be reflected as inattention in ADHD symptoms [46]. Taken together, our results indicate that DCD and ADHD are consistent in that there are problems in properly acquiring sensory information, which is necessary for movement. However, the neurological problems in sensory thresholds associated with motor coordination difficulties (particularly fine motor difficulties) in children with DCD might be different from children with ADHD.

Our results also showed an association between tactile processing problems and fine motor coordination difficulties in children with DCD. This finding is in agreement with a previous study that showed a significant association between the tactile system and self-care skills in children with DCD [18]. In addition, our results showed an association between auditory processing problems and poor general coordination. These associations in the DCD group also differed from that in the TD group. The DCDQ general coordination consists of items about learning new motor tasks, doing daily activities quickly and competently, and maintaining the posture for a long time. These items seem to reflect important functions of the cerebellum, such as motor learning, postural reflexes, and control of independent limb movements, particularly rapid, skilled movements [36, 47]. This association might emphasize that there is a common abnormality in the cerebellum related to mechanisms of motor coordination difficulties and auditory processing problems in DCD.

### Limitations

There are limitations to the present study. First, our sample size was small to medium, which might limit the statistical power to detect some findings. Second, the cross-sectional nature of this study prohibited an exploration of longitudinal interactions between sensory and motor functions and challenges. Third, data obtained through the SP, which is a questionnaire, likely provided us with limited information about the child’s sensory problems. Further studies using direct behavioral observations are required to further elucidate the association between sensory and motor functions and challenges that children with DCD face.

## Summary

This is the first study reporting the sensory processing profiles and the associations of sensory processing problems with motor coordination difficulties in preschoolers with DCD diagnosed through screening and a subsequent in-person assessment in a community sample. Although we excluded ASD and ADHD, our findings in children with DCD were similar to those in previous studies that included other NDDs (particularly ASD). Our findings also indicate that sensory processing abnormalities may contribute to the pathophysiology of DCD, suggesting the importance of assessing sensory processing functions in children with DCD. Further investigations are required to elucidate the neurological mechanism of these sensory processing problems in DCD.

## Electronic supplementary material

Below is the link to the electronic supplementary material.

Supplementary file1 (DOCX 61 kb)
