# Supplementary material for: Atypical Sensory Processing Profiles and Their Associations With Motor Problems In Preschoolers With Developmental Coordination Disorder
Source: Child Psychiatry Hum Dev. 2020 Jun 11;52(2):311–20. doi: 10.1007/s10578-020-01013-5 (PMC7973923; doi:10.1007/s10578-020-01013-5)
Supplement: Supplementary file 1 — Supplementary file1 (DOCX 61 kb) [file 10578_2020_1013_MOESM1_ESM.docx]

Supplemental
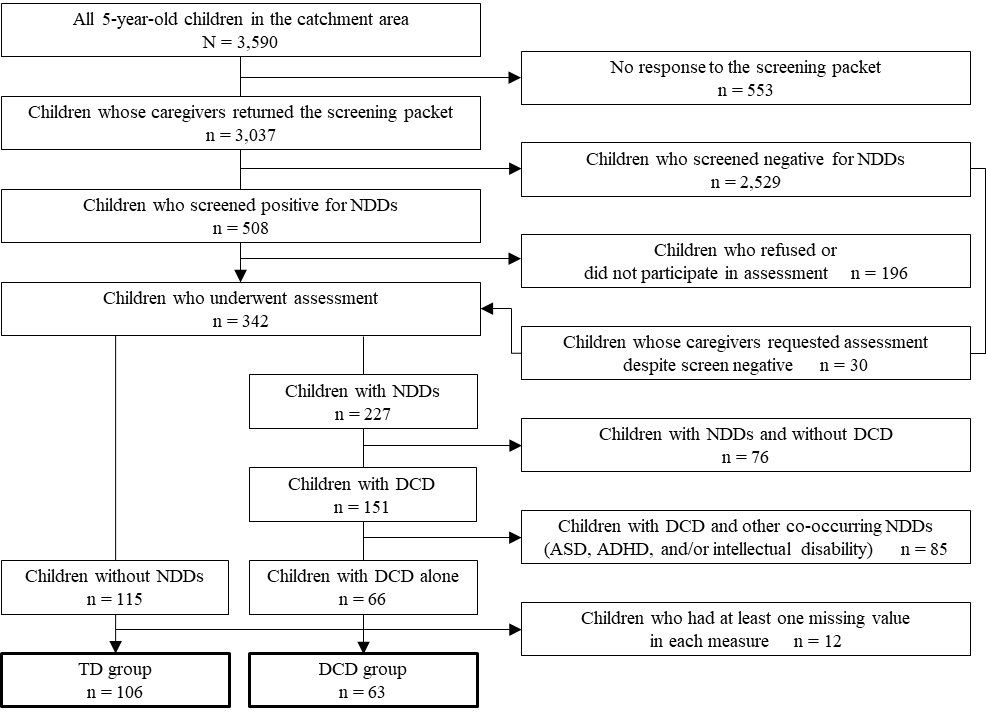
figure. Flow chart of the Hirosaki Five-year-old Developmental Checkup and Assessment. NDDs = neurodevelopmental disorders; ASD = autism spectrum disorder; DCD = developmental coordination disorder; ADHD = attention deficit hyperactivity disorder; TD = typically developing.
